# Predicting the Current and Future Distribution of *Monolepta signata* (Coleoptera: Chrysomelidae) Based on the Maximum Entropy Model

**DOI:** 10.3390/insects15080575

**Published:** 2024-07-29

**Authors:** Qingzhao Liu, Jinyu Zhao, Chunyan Hu, Jianguo Ma, Caiping Deng, Li Ma, Xingtao Qie, Xiangyang Yuan, Xizhong Yan

**Affiliations:** 1 College of Plant Protection, Shanxi Agricultural University, Jinzhong 030800, China; qzliu98@163.com (Q.L.); zhaojinyu312@163.com (J.Z.); hucy4216@163.com (C.H.); mjg7775@163.com (J.M.); mali890310@126.com (L.M.); qxt123@nwafu.edu.cn (X.Q.); 2 College of Forestry, Shanxi Agricultural University, Jinzhong 030800, China; forestdeng99@126.com; 3 College of Agriculture, Shanxi Agricultural University, Jinzhong 030800, China

**Keywords:** *Monolepta signata*, MaxEnt model, potential distribution, climate change, crop insect pests

## Abstract

**Simple Summary:**

*Monolepta signata* is a highly destructive agricultural pest that causes significant economic losses to important economic crops such as maize and cotton in its native area: Asia. This study uses the maximum entropy model, combined with bioclimatic variables and altitude, to predict the potentially suitable areas and spread patterns of *M. signata* worldwide. The research results show that, in addition to its native area, *M. signata* has potentially suitable areas all over the world. The potential geographical distribution of this pest is gradually expanding globally. By predicting the potential occurrence and spread areas of *M. signata* worldwide, this study provides an important theoretical basis for formulating effective prevention and control measures and early-warning strategies for this pest.

**Abstract:**

*Monolepta signata* is a polyphagous and highly destructive agricultural pest, currently only distributed in Asia. In its place of origin, it poses a serious threat to important economic crops, for instance, maize (*Zea mays* L.) and cotton (*Gossypium hirsutum* L.). Based on morphological and molecular data research, it has been found that *M. quadriguttata* (Motschulsky), *M. hieroglyphica* (Motschulsky), and *M. signata* are actually the same species. This discovery means that the range of this pest will expand, and it also increases the risk of it spreading to non-native areas worldwide. It is crucial for global agricultural production to understand which countries and regions are susceptible to invasion by *M. signata* and to formulate corresponding prevention, control, and monitoring strategies. This study uses the maximum entropy model, combined with bioclimatic variables and elevation, to predict the potentially suitable areas and diffusion patterns of *M. signata* worldwide. The results indicate that in its suitable area, *M. signata* is mainly affected by three key climatic factors: Precipitation of Wettest Month (bio13), Mean Temperature of Warmest Quarter (bio10), and Temperature Seasonality (bio4). Under the current status, the total suitable region of *M. signata* is 252,276.71 × 10^4^ km^2^. In addition to its native Asia, this pest has potentially suitable areas in Oceania, South America, North America, and Africa. In the future, with climate change, the suitable area of *M. signata* will expand to high-latitude areas and inland areas. This study found that by the 2070s, under the SSP5-8.5 climate scenario, the change in the potentially suitable area of this insect is the largest. By identifying the potentially suitable areas and key climatic factors of *M. signata*, we can provide theoretical and technical support to the government, enabling them to more effectively formulate strategies to deal with the spread, outbreak, and invasion of *M. signata*.

## 1. Introduction

*Monolepta signata* is a widely distributed polyphagous pest that damages various economic crops such as maize (*Zea mays* L.), cotton (*Gossypium hirsutum* L.), sorghum (*Sorghum bicolor* L.), and potato (*Solanum tuberosum* L.) [1]. In China, *M. signata* mainly damages the leaves and silks of maize during the filling stage, causing yield loss and quality decline, thus ultimately affecting maize production [2]. *M. signata* feeds on cotton bracts, corollas, etc., causing the corolla incomplete stamen to be exposed, affecting pollination, and causing a decline in cotton yield [3]. In addition, *M. signata* has a long period of harmfulness, and short-distance migration characteristics, and will continue to migrate and harm maize and cotton [4,5]. Currently, the global distribution of *M. signata* is expanding, resulting in significant damage to maize productivity and quality, especially in countries such as China, India, Thailand, and Myanmar [6,7,8,9]. Consequently, it has emerged as a significant pest in maize production areas in Asia [9,10,11].

In 2012, based on male genital characteristics and external morphology, *Monolepta hieroglyphica* (Motschulsky) was confirmed to be a synonym of *M. signata* (Oliver) [12], and *Monolepta quadriguttata* (Motschulsky) was confirmed to be a synonym of *M. signata* over 100 years ago [13]. By 2023, researchers had further confirmed them to be the same species through molecular data [14]. Following the identification of *M. signata*, *M. quadriguttata*, and *M. hieroglyphica* as the same species, it means that the range of this pest will expand, and it also increases the risk of it spreading to non-native areas worldwide.

Globally, the number of invasive species introduced by human activities is increasing rapidly [15]. Global warming may increase the metabolic rate of insect pests, thereby increasing the number of harmful insects, and causing more serious losses to agricultural production [16]. Both invasive species and climate change are posing threats to global food security and biodiversity [17]. To prevent its further spread, the first step should be a detailed study of its potentially suitable areas worldwide. However, current research on *M. signata* mainly focuses on its occurrence and harmfulness [18], biological control [19], control efficacy [10], biological characteristics [14], and phylogeny [20]. There is no research on how climate change affects the geographical dimension of *M. signata*. This study will help establish early warning strategies for *M. signata* worldwide to prevent potential losses caused by its further spread.

The methods for evaluating the suitable areas of species include the random forest (RF) [21], geographically weighted regression (GWR) [22], generalized linear model (GLM) [23], and maximum entropy model (MaxEnt) [24]. MaxEnt is based on the maximum entropy theory, with species distribution data and climate variables as the foundation, and simulates the potential geographical distribution range of species through mathematical models [25]. According to the maximum entropy theory, when there is no external interference, entities strive for maximum freedom within constraints [26]. Under known conditions, the state with maximum entropy is most likely to approximate the true state [27]. Therefore, the goal of maximum entropy statistical modeling is to select the distribution with maximum entropy from those that satisfy the given constraints as the optimal distribution [26,27]. The MaxEnt model has many advantages, such as short run time, ease of use, and high simulation precision [28,29,30]. The MaxEnt model is easy to use, requiring only minor adjustments to parameters and settings [26]. In cases where data on the target species’ distribution were limited, this model’s predictive ability surpasses that of many similar models [31]. It is widely used to protect endangered species, identify areas suitable for invasive species, and monitor changes in the ranges and areas of pests such as *Monochamus carolinensis* (Olivier) [24], *Diaphorina citri* (Kuwayama) [32], and *Halyomorpha halys* (Stål) [33]. Therefore, it is a suitable tool for studying the ecological niche of *M. signata*.

This study is based on the existing distribution of *M. signata*. Using the MaxEnt model, the current study predicts the potentially suitable areas of *M. signata* under current and future climate change worldwide. Also, it identifies the principal climate factors that limit the distribution of *M. signata*. The current climate data are predicted based on historical data from 1970 to 2000. The future data selected are SSP1-2.6, SSP2-4.5, and SSP5-8.5 under the Beijing Climate Center Climate System Model version 2 (BCC-CSM2-MR) climate model in Phase 6 of the Coupled Model Intercomparison Project (CMIP6) [34] and chooses two time periods: 2041–2060 and 2061–2080. This research offers a theoretical basis for the early warning and prevention of *M. signata*.

## 2. Materials and Methods

### 2.1. Collection and Screening of Sample Data

**Figure 1:**
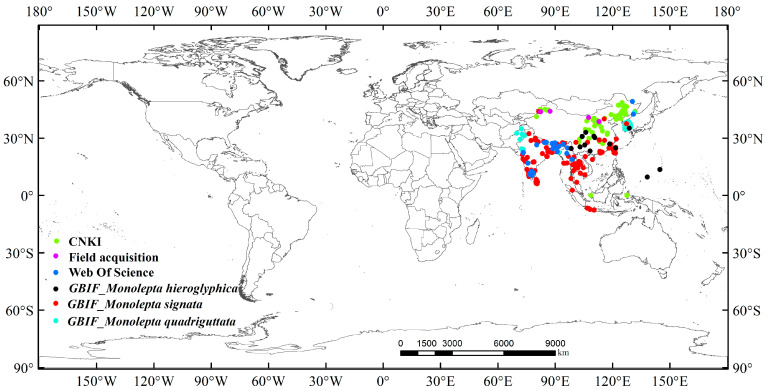
The occurrence data of *M. signata* (different colors represent different distribution sources).

In this study, we collected 1077 distribution data points for *M*. *signata*, a highly destructive agricultural pest. The distribution data of *M. signata* were sourced from official websites and databases. Initially, we entered the Latin names *M. hieroglyphica*, *M. quadriguttata*, and *M. signata* into the Global Biodiversity Information Facility database (GBIF) (accessed on 2 October 2023, at https://www.gbif.org/), obtaining 972 distribution data points. In addition to GBIF, we also collected a total of 105 distribution data points for *M. signata* from the National Specimen Information Infrastructure (accessed on 2 October 2023, at http://www.nsii.org.cn/), the China National Knowledge Infrastructure (CNKI; accessed on 2 October 2023, at https://www.cnki.net/) database, the Web of Science (https://www.webofscience.com/wos/, accessed on 2 October 2023), and field research conducted from 2022 to 2023, using *M. hieroglyphica*, *M. quadriguttata*, and *M. signata* as keywords. We summarized the detailed occurrence of *M. signata* from the results obtained (Figure 1).

To reduce redundancy in the occurrence data [35], we used ArcGIS 10.8.1 software to filter the occurrence data for *M. signata*. We established a buffer zone of 10 km, and any data points less than 10 km apart were expurgated from the dataset. In addition, we removed unreasonable data on the map, such as duplicates, invalid entries, densely clustered points, and latitude–longitude information recorded on GBIF but not reported in this country. Ultimately, we obtained 253 occurrence points for *M. signata*, which is sufficient for constructing species distribution models (Figure 1).

**Figure 2:**
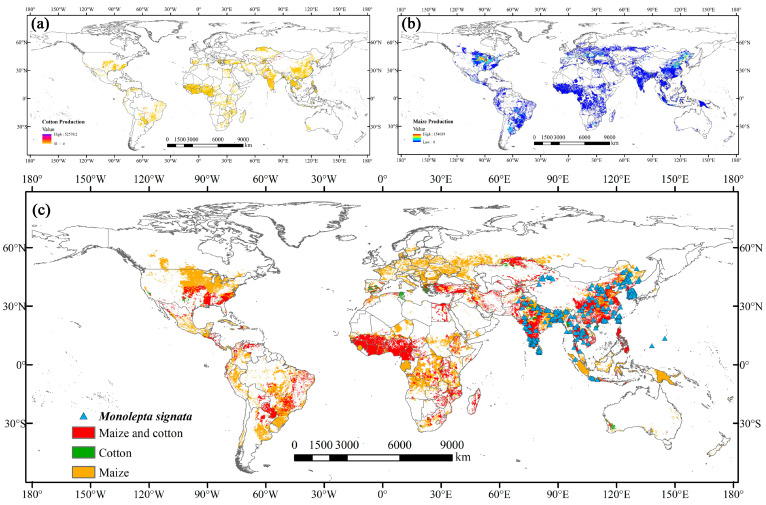
Distribution data for *M. signata* and the production of cotton and maize. (**a**) The areas of cotton production worldwide. (**b**) The areas of maize production worldwide. (**c**) Worldwide occurrence data of *M. signata* and the main host plants.

The production area data for the primary hosts of *M. signata*, cotton and maize, were sourced from MapSPAM2010 (http://mapspam.info/, accessed on 23 July 2024) and further processed using ArcGIS 10.8.1 software (Figure 2).

### 2.2. Environmental Variables

The environmental data were downloaded from the WorldClim database (https://worldclim.org/download from 20 May 2023). The environmental factors included current (1970–2000) and future (2041–2060, 2061–2080) climate data, encompassing 19 bioclimatic variables and elevation data. This study used version 2.1 of the WorldClim database, with the current climate data time span being 1970–2000 and a spatial resolution of 2.5 arc-minutes. The future data are generated on the CMIP6 under the BCC-CSM2-MR model and the three scenarios include SSP1-2.6, SSP2-4.5, and SSP5-8.5 [36]. Compared to CMIP5, the current CMIP6 models exhibit stronger warming due to higher climate sensitivity in the new generation of climate models and updated specifications for concentration, emissions, and socioeconomic development [37]. Meanwhile, BCC-CSM was frequently used to simulate the climate response to elevated greenhouse gas concentrations globally, and its performance is satisfactory [38]. Therefore, using CMIP6 can improve the accuracy of our model predictions. In the three shared socioeconomic pathways (SSPs) chosen for this study, SSP1-2.6 corresponds to a low forcing scenario, SSP2-4.5 represents a medium forcing scenario, and SSP5-8.5 represents a high forcing scenario. These scenarios assume that by 2100, radiative forcing stabilizes along paths of approximately 2.6, 4.5, and 8.5 W/m^2^, respectively [39].

**Figure 3:**
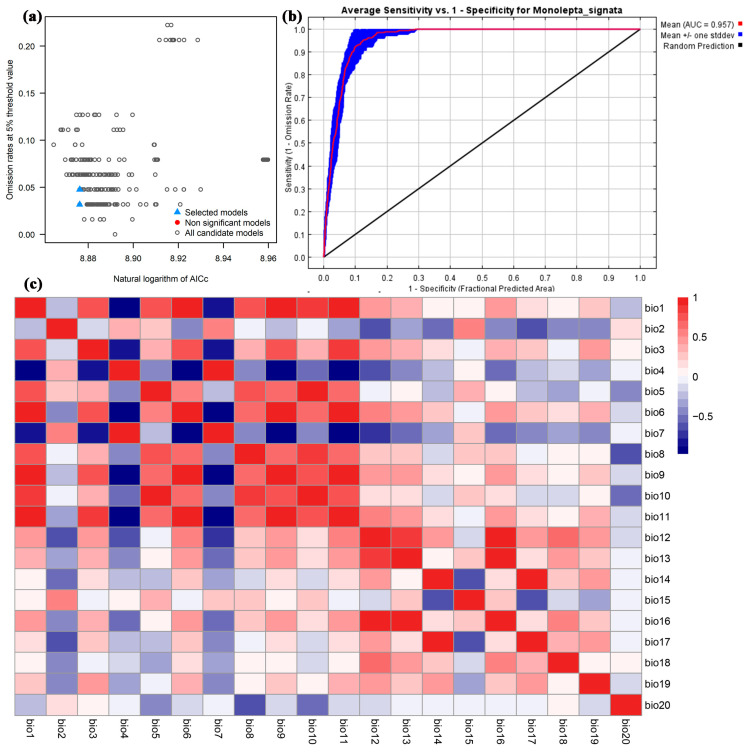
(**a**) The AIC value of the parameter combination (FC, RM) is calculated based on the Kuenm of *M. signata*. (**b**) The ROC curve predicts the distribution of *M. signata*. (**c**) The Pearson correlation coefficient of environmental data for *M. signata*.

The environmental variables provided by WorldClim may be correlated with each other. Multicollinearity is one of the potential causes of overfitting in the model [40]. Therefore, after running the model, we performed a correlation analysis on the environmental variables. For factors with a correlation coefficient r ≥ 0.7 [32], we only retained one based on the contribution rate obtained by the jackknife method. Finally, we selected nine variables to import into the model, namely, Mean Diurnal Range (bio2), Temperature Seasonality (bio4), Mean Temperature of Warmest Quarter (bio10), Precipitation of Wettest Month (bio13), Precipitation Seasonality (bio15), Precipitation of Driest Quarter (bio17), Precipitation of Warmest Quarter (bio18), Precipitation of Coldest Quarter (bio19), and Elevation (bio20) (Figure 3c).

### 2.3. Model Establishment and Optimization

We used MaxEnt 3.4.3 to forecast the potentially suitable area of *M. signata* worldwide. The two key parameters of MaxEnt are FC (feature combination) and RM (regularization multiplier) [24]. FC has five options: linear features (L), quadratic features (Q), product features (P), threshold features (T), and hinge features (H), generating 29 different combinations. The regularization multiplier ranges from 0.5 to 4, setting a value every 0.5, for aggregate 8 RM values. We used the Kuenm package in R, combined with MaxEnt, to perform predictive calculations for 232 different parameter models (free combination of 29 FC settings and 14 RM values) [41]. First, R 4.4.0 software selects a set of statistically significant models with an omission rate ≤ 5% from all candidate models. Then, according to the Akaike Information Criterion (AICc), the model with the comparison value of the optimal model and the existing model AICc (Delta AICc) ≤2 is selected as the recommended model. If R software selects multiple recommended models, we will choose the model with the smallest Delta AICc value as the optimal model [42]. Finally, we used the AICc indicator to screen the parameter combinations of the MaxEnt model and select the combination with the smallest AICc value. Regarding the MaxEnt model, when using approximately 10,000 background pseudo-absences, predictive performance is typically better [26]. Therefore, this research set the maximum of 10,000 pseudo-absences background points and ensured that these sampling points had no missing environmental layers.

### 2.4. Suitable Area Prediction

Under the current climate conditions, we set the following parameters for the model prediction of *M. signata*: We chose “Cloglog” as the output format and “asc” as the file type and used 25% of the distribution points as the random test percentage. We selected “Subsample” as the method for replicating run options and activated the random seed, which ensures that a different random seed will be utilized for each run. We set the number of repetitions to 10 times to reduce the uncertainty caused by outliers. All other parameters were kept at their default settings. These settings are designed to improve the accuracy and stability of model prediction [43].

When predicting the model parameters of *M. signata* under future climate conditions (2041–2060, 2061–2080), we imported the corresponding climate data of SSP1-2.6, SSP2-4.5, and SSP5-8.5 into the projection layer directory of the MaxEnt model. Apart from this, all other parameter settings remained consistent with those under the current climate conditions.

In the final suitable area map for *M. signata*, the values range from 0 to 1, representing the probability of *M. signata* occurring worldwide. The natural breaks (Jenks) method is a convenient and efficient data classification method, which can determine the best arrangement of values in different categories [44,45]. It is used to distinguish the suitable and unsuitable areas for *M. signata* in the final model, and further divides the suitable areas for *M. signata* into four categories: unsuitable (0–0.090); low suitability (0.090–0.309); medium suitability (0.309–0.625); high suitability (0.625–1). We used the raster layer attribute table to calculate the proportion of different suitable areas and, finally, we calculated the area size of different suitable areas.

Multivariate environmental similarity surfaces (MESS) analysis was utilized to examine potential climates that are not represented in the model’s training data. It compares the environmental data from the model’s training with the data from each grid cell in the new projection area to assess their similarity. This analysis was particularly useful for identifying regions that fall outside the environmental range of the training area. The climate similarity across different regions and time periods was determined by the MESS values, where negative values indicate non-analog climate conditions [46].

### 2.5. Accuracy of the Prediction Results of the MaxEnt Model

The accuracy of the MaxEnt model prediction results is tested using the omission rate, the area under the curve (AUC) of the receiver operating characteristic (ROC) curve, and the true skill statistics (TSS) [24]. If the test omission rate is closer to the theoretical omission rate, it indicates that the accuracy of the constructed model is higher. If the test omission rate is higher or lower than the theoretical omission rate, it indicates spatial autocorrelation in the modeling data. When evaluating the prediction results of the MaxEnt model with the AUC value of the ROC curve, in addition to comparing the size of the AUC value, the curve is also important. If the ROC curve extends to the upper left, it indicates that the model prediction sensitivity is higher and the results are more accurate. The range of AUC values is 0–1 [42]. The standard for evaluating the accuracy of MaxEnt model prediction results using AUC values is as follows: if 0 < AUC ≤ 0.6, the prediction result fails; if 0.6 < AUC ≤ 0.7, the prediction result is poor; if 0.7 < AUC ≤ 0.8, the prediction result is general; if 0.8 < AUC ≤ 0.9, the prediction result is good; if 0.9 < AUC ≤ 1, the prediction result is very good [47]. The value of TSS ranges from −1 to +1, where ≤0 means that the model’s performance is not better than random and +1 means that it is completely consistent [48].

## 3. Results

### 3.1. Modeling Performance

Based on the Kuenm package shown in Figure 3, the model optimization suggested that the RM equals 1.5 and FC incorporates “LQPH” for *M. signata* (Figure 3a). The values of the test AUC from the optimized model are shown in Figure 3. The AUC value of *M. signata* was 0.957 ± 0.009 (Figure 3b). The TSS value of *M. signata* was 0.839 ± 0.024. These results clearly show a high performance of the models in predicting the suitable area of *M. signata*.

### 3.2. Contribution Analysis of Environmental Variables

The MaxEnt model prediction results indicate that the variables that significantly influence the potential *M. signata* suitable area are bio13, bio10, bio4, bio15, bio18, bio2, bio17, bio20, and bio19, with contribution rates of 55.3%, 12.2%, 9.9%, 7.8%, 5.1%, 4.2%, 3.7%, 1%, and 0.9%, respectively (Table 1). Among these, bio13, bio10, and bio4 have the greatest impact on the suitable area of *M. signata*, and the cumulative contribution rate of these variables has reached 77.4%. In addition, the permutation importance of bio13, bio10, bio4, bio15, bio18, bio2, bio17, bio20, and bio19 are 1.7, 56.7, 6.9, 2.1, 15.5, 8.3, 4, 3.6, and 1.1, respectively (Table 1).

**Figure 4:**
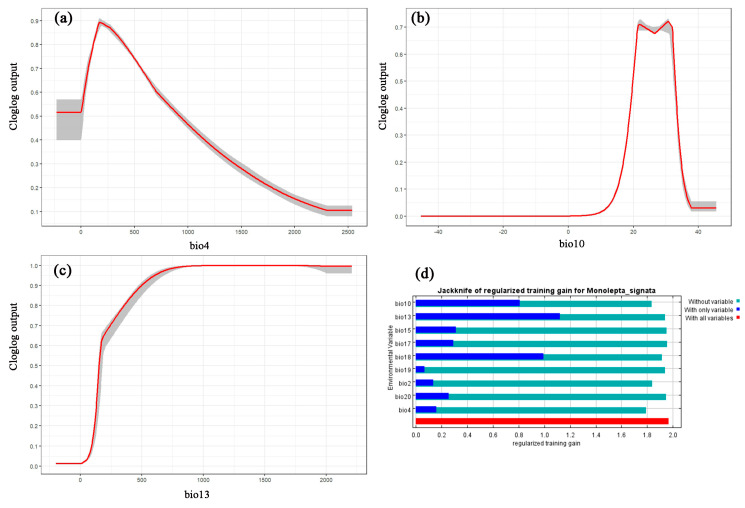
(**a**–**c**) Response curves of *M. signata* to the environmental variables with the highest contribution to model building. (**d**) Jackknife of regularized training gain for *M. signata*.

The jackknife method is a statistical approach used to evaluate the significance of variables in a predictive model. It is particularly useful in predicting species distribution. Optimizing the model through the jackknife method can make the model’s predictions more accurate. We employed this approach to assess the importance of each environmental factor in predicting the distribution of *M. signata*, eliminating those with minimal impact, and then rebuilding the model with the remaining factors. In this process, the length of the blue bars reflects the extent of the variable’s impact on the distribution of *M. Signata*. The shortness of the green bars indicates the richness of the unique information contained in the variable, as well as its potential influence on the suitable area for *M. signata* (as shown in Figure 4d) [29]. The results of the jackknife test reveal that among the nine determined environmental factors, the environmental variable with the highest gain when used alone is bio13. When we ignore bio4, the gain decreases the most, which indicates that bio4 seems to contain information that other variables do not have. Overall, the potential distribution of *M. signata* is mainly affected by three environmental variables (bio13, bio10, and bio4). The suitability value is unimodal, with bio13, bio10, and bio4 being the key environmental variables. Under the condition of the probability of *M. signata* survival and reproduction exceeding 0.5, the response ranges of the key environmental factors are bio13 (>151.55 mm), bio10 (19–32 °C), and bio4 (≤923.70) (Figure 4a–c).

### 3.3. Current Potentially Suitable Region

**Figure 5:**
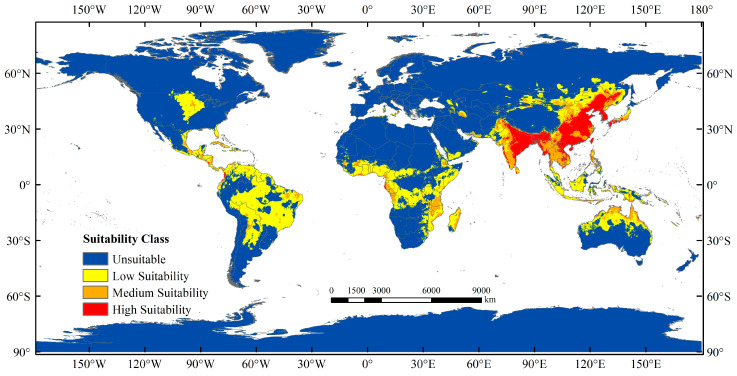
Current potentially suitable area for *M. signata* predicted by MaxEnt model.

**Figure 6:**
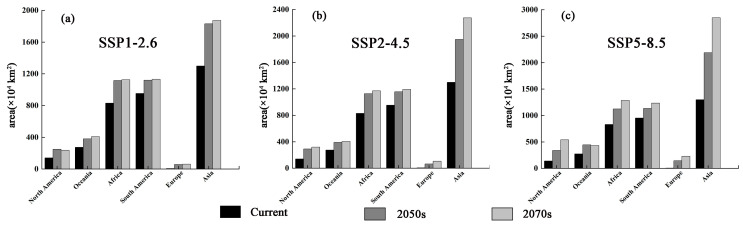
Comparison between the predicted suitable areas (×10^4^ km^2^) of the potential *M. signata* distribution under different current and future climate scenarios.

The predictions obtained using the MaxEnt model for *M. signata* were consistent with the known distributions of *M. signata* (Figure 1 and Figure 4). The potentially suitable areas for *M. signata* worldwide mainly include eastern and southern Asia, northern Oceania, central and southern North America, northern and central South America, and central Africa. The approximate range spans from 60° N to 40° S. Among these regions, Asia has the largest potentially suitable area (Figure 4). The total suitable area for *M. signata* globally is approximately 252,276.71 × 10^4^ km^2^, with high, moderate, and low suitability areas covering approximately 40,695.15 × 10^4^ km^2^, 50,657 × 10^4^ km^2^, and 160,924.51 × 10^4^ km^2^, respectively (Figure 5 and Figure 6).

### 3.4. Potential Suitability Regions Change for M. Signata under Future Climate Scenarios

**Figure 7:**
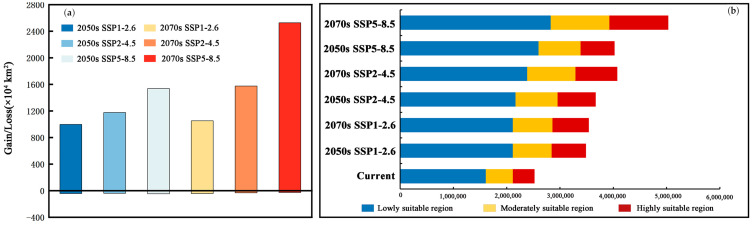
(**a**) Comparison of area changes between the current distribution and the future climate scenario. (**b**) Suitable area under different climatic scenarios.

**Figure 8:**
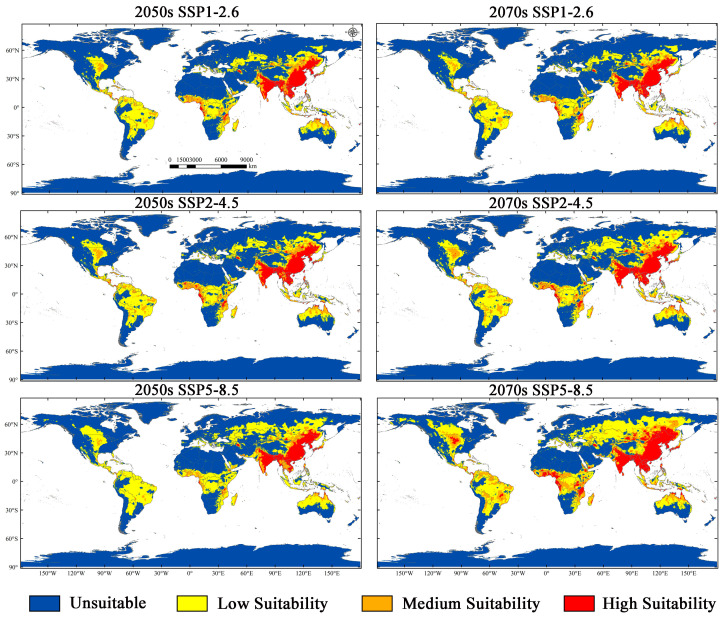
Future potential *M. signata* distribution regions under three climate scenarios (SSP1-2.6, SSP2-4.5, and SSP5-8.5).

The results of the MaxEnt model under three scenarios incorporate SSP1-2.6, SSP2-4.5, and SSP5-8.5 for the 2050s and 2070s, indicating that the total suitable regions for *M. signata* will continue to expand. Under the SSP1-2.6 scenario, by the 2050s, the potential distribution area of *M. signata* is expected to increase by 177.30% compared to the current potentially suitable area. By the 2070s, it will further increase by 181.57%. Under the SSP2-4.5 scenario, by the 2050s, the total area of the potentially suitable area for *M. signata* is projected to increase by 191.93% under the SSP2-4.5 scenario. The potentially suitable area growth rate is expected to be 224.12% by the 2070s. Under the SSP5-8.5 scenario, the potential distribution area of *M. signata* is anticipated to grow by 219.96% by the 2050s, reaching a maximum increase of 300.09% by the 2070s (Figure 7 and Figure 8). Under the six future scenarios, the potentially suitable area of *M. signata* in the world continuously increases. Among them, the area of highly suitable zones has the largest growth rate. By 2070, under the SSP5-8.5 scenario, the highly suitable region is 110,688.10 × 10^4^ km^2^, which is 153.31% higher than the current climate scenario’s high-suitability zone area of 40,695.15 × 10^4^ km^2^ (Figure 7b and Figure 8).

**Figure 9:**
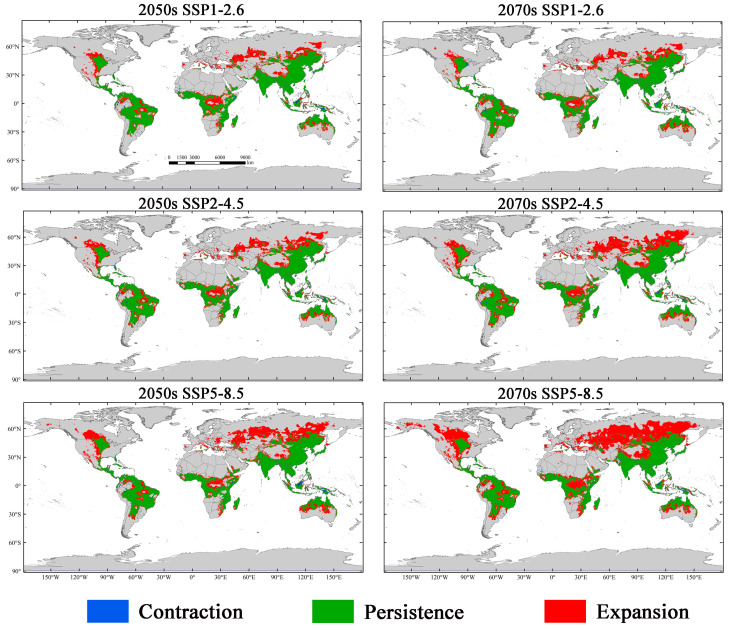
The potential distribution range of *M. signata* under future climate scenarios (SSP1-2.6, SSP2-4.5, and SSP5-8.5) compared with the current potential distribution.

According to the changes in the potentially suitable area under future climate scenarios, the potential distribution area of *M. signata* exhibits significant trends. Under future climate scenarios, the potentially suitable area for *M*. *signata* varies across different continents. Among them, Asia experiences the most significant changes in potential distribution area, followed by South America and Africa. In contrast, Europe shows the smallest variation in potential distribution area. In Asia and North America, *M. signata* suitable areas are expanding northward. In South America and Oceania, the potentially suitable area for *M. signata* is expanding southward. Meanwhile, in Africa, the expansion trend of *M. signata* extends from the coastal areas of central Africa to the inland regions. Under the SSP5-8.5 climate scenario, *M. signata* has the largest expansion area globally (Figure 6 and Figure 9).

### 3.5. Potential Suitability Regions Change of M. Signata under Future Climate Scenarios

**Figure 10:**
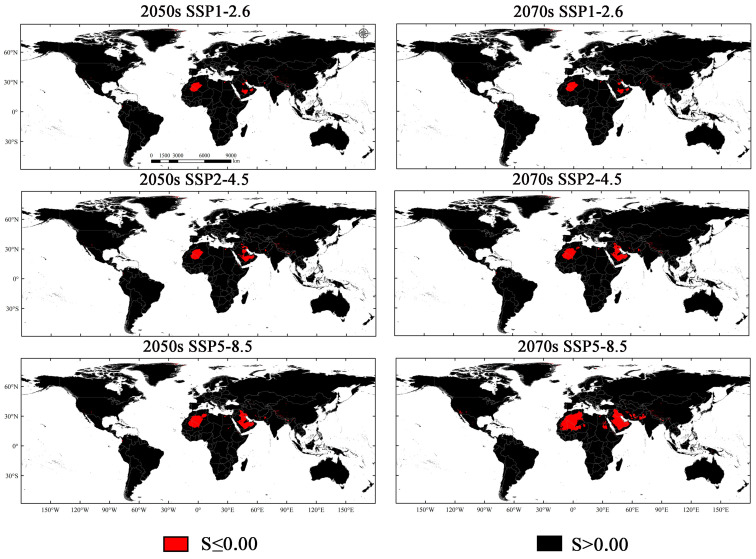
MESS maps for *M. signata* were obtained using the BCC-CSM2-MR for SSP1-2.6, SSP2-4.5, and SSP5-8.5 in scenarios based on the 2050s and 2070s.

The MESS analysis identified environmental conditions that exist within the model’s calibration regions but are absent in the model’s projection areas (Figure 10). According to the results of the MESS analysis, most projection areas share a medium-to-high degree of environmental similarity with countries within the training area, with some exceptions. Under six climate scenarios, these exceptional regions are primarily concentrated in northwest Africa, the Middle East, and East and South Asia, as well as certain regions in North America, Oceania, and South America. Currently, these regions do not have the presence of *M. signata*, but over time, parts of these areas may gradually become suitable areas for *M. signata*. Therefore, future research may need to reassess the potential suitability of these regions (Figure 10).

## 4. Discussion

In this research, we used the MaxEnt model to forecast the potentially suitable areas for *M. signata* globally under climate change. Although there are existing studies on the suitable area of *M. signata* in countries such as China, Russia, Korea, India, and Pakistan, these studies are mostly focused on a smaller scale [14,49,50]. There is still a lack of research on the geographical spread of *M. signata* and the changes in their ecological niches on a larger scale under the influence of climate change. Especially in studying morphological characteristics and molecular data, researchers have confirmed that *M. quadriguttata*, *M. hieroglyphica*, and *M. signata* are the same species [12,13,14]. Therefore, we integrated the distribution data of these species globally to conduct a more accurate study of the suitable areas for *M. signata* worldwide. In this context, we used the MaxEnt model to predict the potentially suitable areas for *M. signata* and determine the range where *M. signata* may occur now and in the future. These predictions are beneficial for the government’s financial input in the prevention and control of *M. signata*. These predictions assist in two ways: one helps the government to timely control areas that have been reported to be harmed by *M. signata* and the other aids in the regular monitoring of areas that have not yet reported any harm from *M. signata* to prevent possible invasion.

In any ecological niche model, the inputting of the target species’ distribution data is a crucial component [51]. Special care is needed when selecting and processing the distribution data of the target species. Increasing the sample data size for the target species and expanding the coverage will result in a greater abundance of species distribution and environmental information that may be obtained. This allows us to establish more constraints for the model, improving the accuracy of potentially suitable area predictions for the species [52]. We conducted a spatial thinning analysis on the 1077 collected distribution points of *M. signata*, ultimately obtaining 253 valid data points. This method effectively reduced the risk of model overfitting [53]. The worldwide increase in *M. signata* distribution data further improved the accuracy of the prediction results, especially in proving that *M. quadriguttata* and *M. hieroglyphica* are synonyms of *M. signata*. This fundamentally reduced the bias in model prediction results caused by a lack of distribution data. In addition to perfecting the distribution data, future data are generated based on CMIP6 under the climate model [54]. This model has been proven to perform well in many aspects of many studies [55], enhancing the accuracy of this prediction of the future suitable area for *M. signata*. Furthermore, this study used the Kuenm package in R to majorize the MaxEnt model parameters to avoid the impact of model overfitting on the prediction results [41].

In the process of predicting the potential area of *M. signata*, we considered environmental factors such as temperature, precipitation, and elevation. Among the environmental variables included in the final model, bio13 (Precipitation of Wettest Month), bio10 (Mean Temperature of Warmest Quarter), and bio4 (Temperature Seasonality) were identified as key factors influencing the suitable area for *M. signata*, contributing significantly to its distribution. Therefore, both precipitation and temperature play crucial roles in shaping the distribution of *M. signata*. Based on the results, precipitation has the most significant influence on the distribution of *M. signata*, followed by temperature. *M. signata* was mainly distributed in areas with subtropical, temperate, and tropical monsoon climates. These areas have abundant precipitation in summer [56]. Research shows that rainfall directly affects the population of *M. signata* [57]. The occurrence of *M. signata* populations was more severe in years with higher rainfall, a phenomenon that may be related to humidity. For instance, in irrigable land, the emergence time of *M. signata* is approximately 13 days earlier than dry areas, and the severity of infestation is more pronounced [57,58]. Similar studies in India have also found a significant correlation between morning relative humidity and the population of *M. signata* [59]. To some extent, these studies suggest that precipitation is critical to the survival of *M. signata*. Additionally, temperature was another important factor influencing the distribution of *M. signata*. Research has shown that within the temperature range of 19–31 °C, the development rate of the overwintering eggs, larvae, and pupae of *M. signata* will accelerate as the temperature rises [4]. This study indicates that Mean Temperature of Warmest Quarter (bio10) within the range of 19–32 °C was suitable for the survival of *M. signata*, which was consistent with the above results. As the global warming trend continues, average temperatures in various regions continue to rise [60]. The predicted results of this study suggest that the high-suitability zones for *M. signata* are primarily distributed in tropical and subtropical areas, and that the tolerance of *M. signata* to high temperatures will favor their growth and development in these regions [61].

This research made clear that under the current climate circumstances, the potential distribution of *M. signata* is mainly between 60° N and 40° S, especially concentrated in East Asia, but it also appears in other continents. The occurrence of *M. signata* in inland areas independently was relatively rare, and in most cases, they gradually spread from suitable areas in coastal areas to inland areas. This reflects *M. signata*’s selection and adaptation to different areas, as well as the impact of human activities on it, to some extent. High-suitability areas in Asia include southeast China, southern Japan, North Korea, South Korea, the Philippines, Laos, Vietnam, Myanmar, Nepal, Bhutan, western and northeastern India, and Bangladesh. Highly suitable areas in Oceania include very few areas in northeastern Australia. The African region includes parts of western Gabon and southern Cote d‘Ivoire. The high-suitability areas in North America include central United States and southeast Mexico. Central America includes northern Panama and a small part of Nicaragua; areas of high suitability in South America include central Brazil, western Colombia, and, to a lesser extent, western Ecuador. The environmental characteristics of these coastal areas were typically high in temperature and precipitation, which is consistent with our previous research results that temperature and precipitation conditions significantly impact the survival and reproduction of *M. signata.* Across various future scenarios, the distribution of *M. signata* may exhibit different expansion tendency. However, all prediction results consistently indicated that China, Japan, South Korea, Russian, North Korea, India, Nepal, Bhutan, Bangladesh, Laos, northern Vietnam, Myanmar, Thailand, the Philippines, Malaysia, Indonesia, and other Asian regions will consistently provide highly suitable areas for *M. signata*. The developed economy and convenient transportation in these regions are conducive to the occurrence and spread of *M. signata* [56,62]. Therefore, enhancing the monitoring and control efforts in these regions is imperative to prevent further spread of *M. signata*.

Relevant studies indicate that *M. signata* was first discovered in the northern regions of Xinjiang, China, in 1998 [63]. Over time, it has spread to other areas in Xinjiang. Meanwhile, during the period from 1961 to 2019, the average temperature in Xinjiang showed a gradual increase, with more pronounced warming in the northern regions such as the Ili River and Altai Mountains [64,65]. As the temperature rises, the breeding rate of *M. signata* accelerates, which poses a greater threat to other regions in Xinjiang. This suggests that the expansion of *M. signata* in this area may be related to climate warming. The process of *M. signata*’s invasion in Xinjiang was similar to the prediction results of this study. In the current climate scenario (1970s–2000s), the suitable area of *M. signata* in Xinjiang is minimal, but in future climate scenarios, the suitable area gradually expands. The fact that *M. signata*’s invasion in Xinjiang, China, and the severe economic losses it caused proves that all countries in the world, especially those where crops such as cotton and maize are important sources of the agricultural economy, should pay close attention to this pest [11]. In addition to the existing areas, *M. signata* has potentially suitable areas in many coastal countries in North America, South America, Africa, and Oceania; over time, they gradually spread to the northern high-latitude areas of inland countries and regions. This may be due to global warming leading to the expansion of the suitable range for the survival and reproduction of *M. signata* [16]. Therefore, countries and regions in North America, South America, Africa, and Oceania where *M. signata* has not yet appeared should strengthen quarantine measures for the introduction of *M. signata* eggs, larvae, and pupae to prevent the further spread of *M. signata* and its potential threat to agriculture.

Host plants can also affect the spread and dispersion of insects, apart from the effects of climate change [66]. Currently, *M. signata* is mainly distributed in most parts of Asia, closely overlapping with maize and cotton production areas in the region (Figure 2). This confirms the accuracy of our distribution data for *M. signata*. Additionally, under the current climate scenario, potentially suitable areas for the pest also exist in maize and cotton production areas in North America, South America, and Africa, increasing the risk of invasion. In China, *M. signata* primarily damages economic crops as adults [1]. The pest has a single generation per year, with a peak occurrence from July to September, lasting approximately three months [57]. Peak adult eclosion runs from late July to early August, and by mid-August, the pest population reaches its maximum size, making it the most critical period for damage [57]. During this same period, maize and cotton, which are major host plants for *M. signata*, are in their crucial stages, making them highly susceptible to severe pest damage. Under future climate scenarios, temperature and precipitation may further increase [67]. Rising temperatures can accelerate the development of overwintering eggs, larvae, and pupae of *M. signata* [4]. This could lead to a shortened reproductive cycle for the pest. Additionally, potentially suitable areas for maize and cotton are expected to expand due to climate warming [68,69,70]. Combining the shortened reproductive cycle of *M. signata* with the increased suitable area, there is a higher risk of rapid reproduction and further spread of the pest during critical growth stages of maize and cotton.

In this study, we predicted the potential occurrence and spread of *M. signata* globally. These predicted areas exceed the current distribution of *M. signata*, including major corn-producing countries such as the United States and Brazil [71], all of which have persistent suitable areas. Considering the influence of climate change on the distribution of *M. signata* in the main maize production areas worldwide, we urgently need to pay attention and formulate effective control and prevention measures to prevent the current and future spread and harm of *M. signata*. Regarding chemical control, dimethoate is an organophosphorus insecticide with systemic absorption. By drip irrigation treatment, dimethoate can effectively control the harm of *M. signata* to maize [9]. In addition, combining thiamethoxam and drip irrigation can also effectively improve the utilization rate of pesticides and achieve precise prevention and control of *M. signata* [10]. Regarding quarantine, biosecurity surveillance is a crucial element in the early detection of invasive alien species [72]. *M. signata* exhibit a significant attraction towards γ-terpinene and D-limonene, which can be further investigated and combined with traps for the early detection of *M. signata* [73]. In addition, the relevant departments should formulate monitoring and evaluation measures for plant products, derivatives, and by-products related to the host of *M. signata* to prevent the further spread of *M. signata*.

The MaxEnt model has advantages such as limited sample size, short run time, ease of use, and high simulation precision [28,29,30]. *M. signata* has numerous host plants in nature [1,4], which significantly affects its distribution and spread. Therefore, future research should incorporate a comprehensive list of host plants for *M. signata* into the model to further clarify its potential predicted distribution. In addition, it also has unavoidable limitations like other species distribution models [74]. In this study, we considered only temperature, precipitation, and elevation as environment factors. However, these are also influenced by various other biological data (e.g., genetic variation, disease, interspecific competition, human activity, and soil vegetation type) [51,75], which can influence the MaxEnt model’s precision. Therefore, future investigations on the distribution of *M. signata* must consider these attributes in order to enhance the predictive ability of the MaxEnt model.

## 5. Conclusions

In this research, the MaxEnt model demonstrated excellent optimal fitting results. The main environmental factors affecting the suitable area of *M. signata* globally include bio13, bio10, and bio4. High-suitability areas are mainly distributed in coastal countries and regions in Oceania, South America, Africa, North America, and Asia. Under different future climate scenarios, the potential distribution for *M. signata* shows an increasing trend, with the largest increase under the SSP5-8.5 scenario in the 2070s. In addition, the future potentially suitable area for *M. signata* tends to shift towards high-altitude areas and inland countries. The potentially suitable area for *M. signata* indicates its risk of spreading and reproducing worldwide. Therefore, this study provides an important reference for formulating global *M. signata* prevention and monitoring strategies for future climate change.

## Figures and Tables

**Table 1:** Percentage contribution (%) of environmental data that have an impact on the suitable area of *M. signata*.

Variable	Percentage Contribution (%)	Permutation Importance
Annual Mean Temperature (bio1, °C)	-	-
**Mean Diurnal Range (bio2,** **°C** **)**	4.2	8.3
Isothermality (bio3)	-	-
**Temperature Seasonality (standard deviation ×100) (bio4)**	9.9	6.9
Max Temperature of Warmest Month (bio5, °C)	-	-
Min Temperature of Coldest Month (bio6, °C)	-	-
Temperature Annual Range (bio7, mm)	-	-
Mean Temperature of Wettest Quarter (bio8, °C)	-	-
Mean Temperature of Driest Quarter (bio9, °C)	-	-
**Mean Temperature of Warmest Quarter (bio10,** **°C** **)**	12.2	56.7
Mean Temperature of Coldest Quarter (bio11, °C)	-	-
Annual Precipitation (bio12, mm)	-	-
**Precipitation of Wettest Month (bio13, mm)**	55.3	1.7
Precipitation of Driest Month (bio14, mm)	-	-
**Precipitation Seasonality (bio15)**	7.8	2.1
Precipitation of Wettest Quarter (bio16, mm)	-	-
**Precipitation of Driest Quarter (bio17, mm)**	3.7	4
**Precipitation of Warmest Quarter (bio18, mm)**	5.1	15.5
**Precipitation of Coldest Quarter (bio19, mm)**	0.9	1.1
**Elevation (bio20, m)**	1	3.6

The bolds are the variables in the final MaxEnt model of *M. signata*.

## Data Availability

The authors confirm that all data in this paper are available.
